# Collaboration in the fight against infectious diseases.

**DOI:** 10.3201/eid0403.980302

**Published:** 1998

**Authors:** D E Shalala


### Collaboration in the Fight Against Infectious Diseases

**Donna E. Shalala**
U.S. Secretary of Health and Human Services

Two hundred years ago, the U.S. Public Health Service, of which the Centers for Disease Control and Prevention (CDC) is an essential part, began as a humble maritime hospital in New York City. Its mission was simply to stop infectious disease from coming in on ships and spreading across our country. Today, as we celebrate the anniversary of the Public Health Service, another historic event has occurred. One of the great detective hunts of the 20th century came to an end. Scientists at the U.S. Department of Defense confirmed that tissue from a woman's body buried near the Bering Strait contains genetic material from the 1918 Spanish flu virus—the virus that caused the worst pandemic the world has ever known. This discovery will help us map the genetic structure of the microbe that sent a wave of death crashing around the globe 80 years ago.

It is hard to believe today that flu could be so nearly apocalyptic. In just 11 months, at least 24 million people died, and most of humanity was infected. The infected often never knew what hit them; in the morning they felt fine; by night they could be dead—drowned as the lungs filled with fluid. There was no explanation, no protection, no cure. The pandemic produced scenes from a gothic horror novel—but it was all too real. In Philadelphia alone, 11,000 died of the flu in a single month. The dead were left in gutters, and death carts roamed the city in a surreal scene from medieval times. As the deaths mounted all over the world, orderly life began to break down. Schools and churches closed; farms and factories shut down; homeless children wandered the streets; their parents vanished. The acting U.S. Army Surgeon General, Victor Vaughn, calculated that if the pandemic continued its mathematical rate of acceleration, it soon could spell the end of humankind.

But then, as silently, as mysteriously, as quickly as it came, the terror began to fade away. People stopped dying. The victims were buried. Life returned to normal. The great flu was soon pushed off the front pages and out of the public eye. When avian flu first appeared last year, we wondered if perhaps another pandemic had begun. An influenza subtype that had never before produced illnesses or deaths in humans now did. While it appears that the spread of avian flu has halted without the appearance of human-to-human transmission, the danger is far from over because the critical period may be just beginning—this is the start of the traditional flu season in Hong Kong.

The emergence of avian flu points up a broader concern: complacency over infectious disease. It is easy to assume that modern medicine has defeated this enemy once and for all. Our comfort is a natural byproduct of our progress and success—the remarkable breakthroughs in antibiotics and vaccines, thanks to the work of scientists and researchers worldwide. We eradicated smallpox—consigning one of history's deadliest killers from the medical books to the history books. But infectious disease remains the leading cause of death worldwide and the third leading cause in the United States. While we may be winning some old battles, we are struggling with some new adversaries—emerging infectious diseases such as Ebola, hantavirus infection, new strains of tuberculosis (TB), AIDS, and Lassa fever, to name a few. In fact, the World Health Organization (WHO) has labeled the growing threat of infectious disease a global crisis.

The time has come to replace complacency with a new sense of urgency—to launch a renewed, unified, global effort against infectious disease. Nature may have the power to create a pandemic—but together we have the power to prevent it, to stop it, to overcome it, to cure it. And there is no time like the millennium. For today, history and human progress have created an "ironic contradiction" in the fight against infectious disease: some of the same forces that invite pandemics can also be harnessed to fight pandemics. With the globalization of travel and trade, immigration, communication, and industrialization, we have a smaller world with porous borders. Nations are more interconnected, people are more interdependent, and humanity is less divided by what the Indian poet Tagore called our "narrow domestic walls." So the bad news is that we have fewer barriers against the spread of infectious disease; yet the good news is that those fewer barriers mean new avenues to progress and the potential for sharing information and efforts to stop infectious disease.

We now have the power to push infectious diseases off the world stage but only if governments, world health organizations, the private sector, scientists, and researchers work together with a global strategy. How do we successfully wage this global battle against infectious disease? The answer lies in what we can learn from the 1918 pandemic; it provides three important lessons—challenges for all of us.

The first lesson is that we must assume it could happen again. Influenza pandemics have regularly swept the world every 10 to 40 years, and it has been 30 years since the last influenza pandemic, Hong Kong flu, killed 700,000. Nature is creative, and the flu has great potential for mutating. If a strain changes dramatically, we could suddenly have a virus for which we may have no immunity, no vaccine, and no cure. The threat is not just the flu—the spectrum of new infectious diseases is constantly expanding, while old diseases, such as TB, have evolved into entirely new killers because they developed antibiotic resistance.

The advent of antibiotics in the 1940s was one of the chief reasons we began to defeat infectious disease. However, almost as soon as antibiotics were available, microbes mutated and developed resistance. In the 1950s to 1970s, we produced so many new antibiotics that there was always an alternative medication; today, the flood of new antibiotics has diminished to a trickle, while the microbes have continued to grow resistant. Antibiotic-resistant bacteria are becoming more common in hospitals and among patients with depressed immune systems. In Japan in 1996 and in the United States last year, we started to see a strain of staphylococcus infection, the most common hospital-acquired infection, which could sometimes withstand vancomycin—our most potent treatment. But almost simultaneously, the first antibiotic to fight a new generation of "super bugs," Synercid, won limited approval from a Food and Drug Administration (FDA) advisory panel. If it wins full approval, it will be the first drug in a new arsenal of weapons. FDA continues to work with drug manufacturers to bring new antibiotics to market as safely and rapidly as possible.

Antibiotic resistance is not just a medical problem; it is also a behavioral problem. Patients too often demand antibiotics for every illness—even for viral infections (like the flu) that do not respond; patients often do not finish the course of medication, allowing the remaining bacteria to develop resistance; many doctors overprescribe; and the pharmaceutical industry has limited its antibiotic development because of cost. The widespread use of antibiotics in farm animals may also be helping the spread of drug-resistant genes. Given the consequences, we must act now to combat the diminishing effectiveness of antibiotics. That is why CDC is strengthening surveillance and implementing education campaigns about the problem, why the National Institutes of Health (NIH) is studying resistance, and why FDA is promoting judicious antibiotic use. But this is not a job for government agencies alone. Each and every one of us who understands the risks needs to spread the message that antibiotics are being misused, abused, and overused.

The next pandemic could also result not from a mutating bug or ineffective antibiotics but from an act of bioterrorism. Whether bioterrorism is state sponsored or undertaken by a lone terrorist, it is not just a problem for the military or law enforcement; it is also a challenge for the entire public health community. If a specific threat is issued—perhaps someone claims to have released a toxic agent in a public place—trained public health officials must first verify that an incident has occurred. They may need to decontaminate the area, identify exposed populations, and deliver preventive measures and treatments. Too often, a threat is not issued, no warning is given. In such a situation, public health officials must first quickly determine the deadly agent, the route of exposure, and the likely source.

The U.S. Department of Health and Human Services (DHHS) is coordinating with our partners in other agencies and the military to ensure the proper training of state and local health officials, the availability of vaccines and drugs, and the enhancement of our surveillance capacity and expertise. There is also an administrationwide effort to train emergency response teams and health-care providers in 120 cities. We must enhance our ability now to address the growing threat of bioterrorism.

The second lesson concerns preparation for a potential pandemic. We cannot wait until the next deadly microbe appears on the world stage. Therefore, since 1993, HHS has been leading a federal, state, and local effort to develop a "pandemic influenza plan." As a result of the avian flu episode, we have sped up the process to complete the plan and pursue its full implementation. Meanwhile, CDC is studying the impact of antiviral medications and alternative ways to produce vaccines. NIH is working with the pharmaceutical industry to develop and test innovative vaccines, including a nasal spray that delivers an inoculation dose of the virus. FDA is issuing new drug permits for experimental influenza vaccines. With new viruses knocking at the door, we cannot afford to be caught unprepared. Because only in the movies can we save the world from a deadly disease in just 24 hours.

We need commitment in responding to all emerging infectious disease. We need a worldwide "surveillance and response network" that can quickly identify and stop an outbreak. We have already laid the groundwork for such a system with bilateral and multilateral talks on disease monitoring with our partners in Europe, Japan, Asia, and Africa. For example, at the Denver Summit in 1997, the group of eight industrialized nations, including the United States, pledged to help develop a global disease surveillance network and coordinate an international response to infectious disease. Working through the Trans-Atlantic Agenda with the European Union (EU), the United States and EU countries have begun to share surveillance data on Salmonella infections. Additionally, through the U.S.-South Africa Bilateral Commission, our two countries are training health personnel in South Africa in surveillance and applied epidemiology. I look forward to working closely with WHO to further globalize our approach to surveillance and response.

U.S. agencies are already supporting the efforts of WHO to improve communications networks and to build regional centers for monitoring disease. CDC and WHO jointly run 12 world monitoring stations for the flu alone. Perhaps the best example of the kind of monitoring and surveillance system needed worldwide is the excellent system that stopped the avian flu outbreak in Hong Kong. On a routine basis, officials collect throat swabs from people with flulike symptoms. The samples are analyzed, and if suspicious, they are immediately sent to CDC, which functions as one of the WHO International Reference Laboratories for East Asia. When the first known case of avian flu was diagnosed in a 3-year-old boy, warning bells went off immediately. When a second case appeared in November, health officials around the world went on alert, and a team from CDC left for Hong Kong. Over the next 2 months, work continued to define the extent of the outbreak, including who was becoming ill, why they were becoming ill, and whether the virus could spread from person to person and cause a pandemic. The slaughter of more than one million chickens seems to have halted the virus at least for now.

Hong Kong's surveillance system proved that early detection of infectious diseases can prevent their spread. David Heymann of WHO once asked a provocative question: What would have happened if we had had an excellent surveillance system in place in Africa when the AIDS outbreak first occurred? Perhaps we could also have halted that virus in its tracks. Perhaps we would have spared ourselves the second great pandemic of the 20th century. AIDS taught us that regardless of a person's sexual orientation, color, wealth, or home, if we hesitate in our fight against infectious diseases and fail to detect and track them early, they will eventually affect us all.

We cannot simply deal with each potential pandemic as it arises. We must also look over the horizon and seize new possibilities to head off infectious diseases before they can occur. We must fully harness this golden age of global telecommunications (from satellites to the Internet) to create a truly global surveillance and monitoring network and find new ways to prevent, stop, overcome, and cure infectious disease. That is one of the reasons that President Clinton proposed the 21st Century Research Fund—a historic national effort to spur the best minds of this generation to unlock scientific discoveries, unravel scientific mysteries, and uncover scientific advances. Today, the pace of medical discovery is not limited by science or imagination or intellect but by resources. Thus, the research fund will provide a US$1.1 billion budget increase for NIH next year. It is the first down payment on an unprecedented 50% expansion of NIH over the next 5 years. This funding will enable NIH to do more to develop new ways to diagnose, treat, and prevent disease. We are also seeking a boost in CDC funding to step up our ability to identify and investigate infectious disease outbreaks, including foodborne outbreaks. CDC will play a key role in a new initiative by the U.S. Agency for International Development to develop programs in targeted countries to fight the growing threat of bacterial resistance, TB, and malaria. This new American investment in fighting infectious disease will not only pay off in America, because in this world without borders, a discovery by any one nation will benefit us all and brings us a little closer to preventing the next pandemic.

The third lesson of the great pandemic of 1918 is that we have the power to prevent the next pandemic and defeat emerging infectious diseases, but only if our nations step up the fight together. Because diseases recognize no borders, in our fight against them, neither can we. Or as Dr. Bruntland of WHO has stated, when it comes to public health, "solutions, like the problems, have to be global in scope." That is why U.S. and Japanese scientists have held three international conferences together on infectious diseases and research. It is why some members of the Asian-Pacific Economic Cooperation Area, including Thailand, Indonesia, and the Philippines, have developed a communications network to track cases of multidrug-resistant TB. And it is why CDC, FDA, and other U.S. agencies are providing assistance to the Russian Federation and the Newly Independent States, which have faced a large increase in infectious disease in the post-Soviet era.

If we truly want to end the threat of infectious diseases, we must do even more together. We must inject into global gatherings—no matter where they are, no matter what the subject—the urgency of working together to defeat infectious disease. We must never let research into infectious disease become a forgotten step-child. We must continue to invest in vaccine research and development and ensure that preventive vaccines are available, affordable, and effective everywhere. We must work with all our partners in the private sector to ensure that drugs, vaccines, and tests are available during an infectious disease emergency. We must ensure that all urban populations have access to essential facilities, especially clean water, because vaccines and medicines can do little if water is unclean. We must work together to deal with urban overcrowding, poverty, and poor sanitation, which are spreading infectious disease in many parts of the world. Finally, we must pool our greatest resources—our imagination and intellect—to fight this collective fight. For as Joshua Lederberg once noted, "Pitted against microbial genes, we have mainly our wits."

Let us pit our wits (and our will) to this battle, together, to heed the lessons of the great pandemic and so ensure that it does not happen again, that we are prepared, and that we always work together. If we do, our children—the children of the millennium—will remember the 21st century as a time of health and hope, a time of promise and possibility, a time of medical miracles and scientific marvels. I have absolutely no doubt that we can do it, that we must do it, that we will do it.

